# Neuroprotective and Anti-Inflammatory Effects of Linoleic Acid in Models of Parkinson’s Disease: The Implication of Lipid Droplets and Lipophagy

**DOI:** 10.3390/cells11152297

**Published:** 2022-07-26

**Authors:** Jesus Alarcon-Gil, Ana Sierra-Magro, Jose A. Morales-Garcia, Marina Sanz-SanCristobal, Sandra Alonso-Gil, Marta Cortes-Canteli, Mireia Niso-Santano, Guadalupe Martínez-Chacón, Jose M. Fuentes, Angel Santos, Ana Perez-Castillo

**Affiliations:** 1 Instituto de Investigaciones Biomédicas “Alberto Sols” (CSIC-UAM), 28029 Madrid, Spain; alarcon.gil.jesus@gmail.com (J.A.-G.); ana.sm2392@gmail.com (A.S.-M.); jmoral06@ucm.es (J.A.M.-G.); msanz@iib.uam.es (M.S.-S.); sandra.alonso@cib.csic.es (S.A.-G.); 2 Cellular Neurobiology Laboratory, Neurobiology Department, Instituto Ramón y Cajal de Investigaciones Sanitarias, Hospital Ramón y Cajal, Ctra. Colmenar km 9.1, 28034 Madrid, Spain; 3 PhD Program in Neuroscience, Autonoma de Madrid University, 28029 Madrid, Spain; 4 Centro de Investigación Biomédica en Red en Enfermedades Neurodegenerativas (CIBERNED), Instituto de Salud Carlos III, 28029 Madrid, Spain; mcortes@cnic.es (M.C.-C.); mnisosan@unex.es (M.N.-S.); gmartinezchacon@hotmail.com (G.M.-C.); jfuentes@unex.es (J.M.F.); piedras3@med.ucm.es (A.S.); 5 Departamento de Biología Celular, Facultad de Medicina, Universidad Complutense de Madrid, 28040 Madrid, Spain; 6 Centro Nacional de Investigaciones Cardiovasculares (CNIC), 28029 Madrid, Spain; 7 Instituto de Investigación Sanitaria Fundación Jiménez Diaz, 28040 Madrid, Spain; 8 Departamento de Bioquímica y Biología Molecular y Genética, Facultad de Enfermería y Terapia Ocupacional, Universidad de Extremadura, 10003 Cáceres, Spain; 9 Instituto de Investigación Biosanitaria de Extremadura (INUBE), 06006 Cáceres, Spain; 10 Departamento de Bioquímica y Biología Molecular, Facultad de Medicina, UCM, Avda. Complutense s/n, 28040 Madrid, Spain

**Keywords:** autophagy, lipophagy, neurodegeneration, lipid droplets, oxidative stress

## Abstract

Parkinson’s disease (PD) is the second most prevalent neurodegenerative disease after Alzheimer’s disease. The principal pathological feature of PD is the progressive loss of dopaminergic neurons in the ventral midbrain. This pathology involves several cellular alterations: oxidative stress, mitochondrial dysfunction, loss of proteostasis, and autophagy impairment. Moreover, in recent years, lipid metabolism alterations have become relevant in PD pathogeny. The modification of lipid metabolism has become a possible way to treat the disease. Because of this, we analyzed the effect and possible mechanism of action of linoleic acid (LA) on an SH-SY5Y PD cell line model and a PD mouse model, both induced by 6-hydroxydopamine (6-OHDA) treatment. The results show that LA acts as a potent neuroprotective and anti-inflammatory agent in these PD models. We also observed that LA stimulates the biogenesis of lipid droplets and improves the autophagy/lipophagy flux, which resulted in an antioxidant effect in the in vitro PD model. In summary, we confirmed the neuroprotective effect of LA in vitro and in vivo against PD. We also obtained some clues about the novel neuroprotective mechanism of LA against PD through the regulation of lipid droplet dynamics.

## 1. Introduction

Parkinson’s disease (PD) is the second most common neurodegenerative disease after Alzheimer’s disease. The principal hallmarks of PD are the death of the dopaminergic neurons, specifically in the Substantia nigra pars compacta (SNpc), and the presence of gliosis (pathological proliferation of reactive glial cells) [1,2]. However, the pathogeny of PD is still unknown, despite its impact on health, society, and the economy [2,3]. Several cellular alterations play important roles in PD development, for example, oxidative stress, mitochondrial dysfunction, loss of proteostasis, impaired autophagic flux, and endoplasmic reticulum stress [3]. Oxidative stress is provoked by the high energetic demand of dopaminergic neurons, dopamine metabolism itself, as well as mitochondrial dysfunction [4,5]. Moreover, autophagic flux alterations, a loss of proteostasis, and the relationship between both alterations play principal roles in PD development [3]. In recent years, lipid metabolism alterations have also become relevant in PD pathology, including the alteration of lipid droplet dynamics. The modification of lipid metabolism has emerged as a possible therapeutic approach for this disease [6,7].

Lipid droplets (LDs) are cellular organelles formed by a core of neutral lipids surrounded by a phospholipid monolayer with different proteins on their surface, like those of the perilipin family [8]. The main components of these LDs are triacylglycerides which are synthesized in the endoplasmic reticulum by several enzymes, such as the diacylglyceroltransferases (DGAT), [9]. The membrane of the endoplasmic reticulum accumulates these triacylglycerides until reaching a determined concentration. At that time, the membrane of the reticulum expands and curls to give rise to the nascent LDs. Then, the LDs grow up further and ends up separating from the endoplasmic reticulum to be released into the cytoplasm [10]. Later, these LDs can be degraded in two ways: lipolysis and lipophagy. Lipolysis is a type of neutral degradation carried out by various enzymes in the cytoplasm. Lipophagy is an acidic degradation of the LDs accomplished through autophagy, either micro- or macro-autophagy. The latter process starts with the generation of a phagophore that includes all LDs or a portion of them to give rise to a lipoautophagosome. This lipoautophagosome fuses with a lysosome to generate the autolysosome, from which the LDs are degraded [11].

The relationship between PD and lipid metabolism (LD biogenesis and lipophagy) has not been studied in depth. Shimabukuro et al. suggested that neural cells could accumulate LDs together with autophagy markers during the aging process [12]. Other authors have demonstrated that neurons increase lipid production and astrocytes enhance LD accumulation under stressful conditions, possibly due to a neuron–astrocyte coupling involved in lipid metabolism [13,14]. Interestingly, an increasing body of evidence is indicating that LDs could be a sink for free radicals [15,16,17] and, consequently, could play an antioxidant and protective role in the pathogeny of PD. On the other hand, several fatty acids (e.g., linoleic or oleic acid) modify LD levels by regulating the autophagic flux and LD biogenesis [18,19]. Each of these pieces of evidence suggests the importance of LDs in the pathogeny of PD. However, it is necessary to further study the connections between these processes.

Given this background, this research aims to demonstrate the neuroprotective and anti-inflammatory effects of linoleic acid (LA) using in vivo and in vitro models of PD. We also aim to confirm that these effects are due to the stimulation of LD biogenesis and lipophagic flux in our in vitro model of PD. We hypothesize that the increased number of LDs act as scavengers of free radicals. Then, their clearance by enhanced lipophagy could act as an antioxidant mechanism. We name this mechanism the “LD recycler mechanism”.

Our results partially demonstrate this hypothesis. We confirm the neuroprotective and anti-inflammatory effect of LA using in vitro and in vivo models of PD. LA appears to act as an antioxidant, LD biogenesis stimulant, and an inducer of lipophagy. This new mechanism of action for LA opens new avenues for the development of novel treatments against PD.

## 2. Materials and Methods

### 2.1. SH-SY5Y Cell Cultures

SH-SY5Y, a human-neuroblastoma-derived cell line (ATCC (Ref.CRL-2266)), was cultured and propagated with RPMI medium (Sigma-Aldrich, Madrid, Spain, R8758) supplemented with 2 mM of glutamine, 10% fetal bovine serum, and 100 μg/mL of gentamicin.

### 2.2. Cell Culture Treatments

In order to attain 70% confluence, cells were seeded onto 96-well plates (3 × 10^4^ cells/well) for the cell viability evaluation and nitrite measurement, onto 24-well plates (1.0 × 10^5^ cells per well) for the immunocytochemical analysis, and onto 100 mm culture plates (1.0 × 10^6^ cells per well) for the flow cytometer and Western blot assays.

To evaluate the cytotoxicity of LA, cell cultures were treated with LA at different concentrations and incubated in the presence or not of 6-OHDA (35 µM). Stemming from these results, an LA concentration of 25 μM and incubation time of 24 h were chosen as the optimal conditions for the experiments. Some other cultures were treated with PF-06424439 (PF, 40 nM, Sigma) or chloroquine (CQ, 40 μM, Sigma, St. Louis, MI, USA) prior to 6-OHDA exposure. Finally, after 18 h of treatment with 6-OHDA, different analyses were performed.

### 2.3. Cell Viability Assay (MTT)

Cell viability was determined by a colorimetric assay with tetrazolium salt (3-[4,5-dimethylthiazol-2-yl]-2,5-diphenyltetrazoliumbromide; MTT; Roche Diagnostic GmbH, Manmheim, Germany, 11465007001), a method that is based on the ability of viable cells to reduce the MTT to formazan. After exposure to 6-OHDA, cells were incubated with MTT (0.5 mg/mL) diluted in cell medium for 1 h. Then, the medium was removed, and the resulting formazan crystals were dissolved with dimethylsulfoxide (DMSO, Sigma). Finally, the reduced MTT was quantified by 595/650 nm absorbance in a spectrophotometer.

### 2.4. Griess Assay

The nitrite concentration in the cell culture medium was measured by Griess assay. For this purpose, 100 μL of media was mixed with 100 μL of Griess reagent (Sigma, final concentration of 40 mg/mL) and incubated for 15 min at room temperature while protected from light. Finally, the absorbance at 540 nm was measured in a spectrophotometer, and the nitrite concentration was calculated by absorbance data extrapolation using a standard curve with sodium nitrite.

### 2.5. Immunocytochemistry Assay

Cells were seeded on glass coverslips in 24-well cell culture plates precoated with poly-D-lysine (20 μg/mL) and treated as mentioned above. After treatment, coverslips were washed in PBS 1X and fixed in paraformaldehyde (4%) for 15 min. After several rinses in PBS, cells were blocked in PBS containing 0.01% Triton X-100 (Sigma) and 10% goat serum for 30 min at 37 °C and incubated with primary antibodies diluted in PBS with 0.01% Triton X-100 and 5% goat serum for 1 h at 37 °C. The following antibodies were used: α-cleaved caspase 3 (1:500, R&D Bio-Tech., Abingdon, UK, AF835), α-COX2 (1:200, Santa Cruz Biotechnology, Heidelberg, Germany, sc-376861), α-SQSTM1 (p62, 1:300, Cusabio, Houston, TX, USA, CSB-PA615696LA01HU), and/or α-ADRP (1:200, Santa Cruz, sc-377429). Then, after several rinses in PBS, cells were incubated with secondary antibodies diluted in PBS with 0.01% Triton X-100 and 5% goat serum for 1 h at 37 °C. The following antibodies were used: Alexa Fluor 488 (ThermoFisher Scientific, Madrid, Spain; Ex 490 nm/Em 525 nm), Alexa Fluor 568 (ThermoFisher; Ex 578 nm/Em 603 nm), and/or Alexa Fluor 647 (ThermoFisher; Ex 650 nm/Em 665 nm). DAPI (4’,6-diamino-2-phenylindole) was used as a nuclear marker. Finally, glasses were mounted with Vectashield (Vector Labs, Newark, CA, USA, H-1000-10), and images were acquired with a confocal microscope LSM710 (Carl Zeiss Inc, Carl Zeiss Iberia, S.L-Division Microscopy, Tres Cantos, Madrid, Spain) or an epifluorescence microscope (Nikon 90i). Fiji Software 1.53c (plus) [20] and JaCop Plugin [21] were used to carry out the different analyses of the obtained images.

### 2.6. BODIPY 493/503 and BODIPY 581/591 C11 Staining

Staining with BODIPY 493/503 (Sigma, 1 μM) and BODIPY 581/591 C11 (Sigma, 2 μM) was performed prior to fixation or flow cytometry analysis. BODIPY 581/591 C11 was added to the 6-OHDA treatment. BODIPY 493/503 was added to cultures 15 min before finishing the incubation with 6-OHDA. Then, immunocytochemistry or flow cytometry analysis was performed.

### 2.7. Flow Cytometry

After treatments, 1.0 × 10^6^ cells per experimental group were stained with BODIPY 493/503 (Sigma, 1 μM) or BODIPY 581/591 C11 (Sigma, 2 μM) [22]. BODIPY 581/591 C11 was added to the 6-OHDA treatment. BODIPY 493/503 was added to cultures 15 min before finishing the incubation with 6-OHDA. Then, cells were washed with PBS 1X, collected by trypsinization, and diluted in 0.5 mL PBS 1X in flow cytometry tubes. Finally, samples were analyzed in a FACS Canto II flow cytometer, and light emissions at different wavelengths of stained cells were measured (530/30 and 582/15 filters for BODIPY 581/591 C11 and 530/30 filter for BODIPY 493/503). Data from cell aggregates, dead cells, and cell debris signals were removed.

### 2.8. Immunoblot Analysis

After 6-OHDA exposure, cells were washed with PBS 1X, and their proteins were isolated using ice-cold RIPA buffer with protease and phosphatase inhibitors (10 mM sodium pyrophosphate, 1 mM phenylmethylsulphonyl fluoride (PMSF), and 1 mM sodium orthovanadate). Then, a total protein amount of 35 µg was subjected to 12% SDS-PAGE and transferred into PVDF membranes (Protran, Whatman). After blocking with 5% fat-free milk in T-TBS (Tween-20 0.05% and 20 mM Tris-HCl pH 7.5 in distilled water) for 1 h at room temperature, the membranes were incubated with different primary antibodies diluted in the same blocking medium for 18 h at 4 °C. A α-LC3β antibody (1:500, Santa Cruz, sc-376404) was used. As a control, an anti-tubulin (1:2000, Sigma, T5168) antibody was used. Then, the membranes were washed with T-TBS and incubated at room temperature with the corresponding secondary antibody diluted in blocking medium for 1 h at room temperature. Proteins were finally detected by the ECL chemiluminescence system (Amersham) in accordance with the manufacturer’s instructions. Fiji software was used for quantification [20].

### 2.9. Animals

All animal experiments were specifically approved by the “Ethics Committee for Animal Experimentation” of the Instituto de Investigaciones Biomedicas (CSIC- UAM) and carried out in accordance with the European Communities Council Directive (2010/63/EEC) and National regulations (RD1386/2018). The experimental design was planned to minimize the number of animals used and to reduce damage to animals. Adult male C57BL/6 mice (3 months) were housed under standard temperature and humidity conditions with 12-h light–dark cycles and ad libitum access to food and water.

### 2.10. Parkinson’s Disease Animal Model

The PD experimental model is based on the intracerebral injection of 6-OHDA into the Substantia nigra pars compacta (SNpc) of mice. 6-OHDA is the most frequently used neurotoxin to model PD since, as a selective catecholaminergic neurotoxin, it produces specific lesions in the nigrostriatal system with progressive retrograde neuronal degeneration that follows a very similar pattern to that described in PD patients [23].

### 2.11. Treatment of Animals

Treatments were intracerebrally injected in mice using a stereotaxic apparatus (Kopf Instruments, Tujunga, CA, USA, Model 900LS). After anesthesia with a mixture of ketamine (60 mg/kg) and medetomidine (0.5 mg/kg), an intracerebral injection was made unilaterally in the SNpc (according to the coordinates of Paxinos and Franklin, from Bregma: 3.2 mm posterior, 2.0 mm lateral, and 4.7 mm depth) with a micropump. Three animals per experimental group were injected with the vehicle (PBS 1X), LA (16 μg) and/or 6-OHDA (1 μg). After injection, animals were individually housed to recover, and they were sacrificed 7 days after lesioning.

### 2.12. Tissue Processing

After animal perfusion with 4% paraformaldehyde, brains were removed, postfixed overnight in the same solution at 4 °C, cryoprotected in 30% sucrose, frozen, and finally, coronal sections of 30 μm thickness were obtained in a cryostat (Cryocut 1900, Leica Biosystems, Barcelona, Spain, CM1900).

### 2.13. Immunohistochemical Assays

Brain sections containing the SNpc were used to perform the immunohistochemical assays. These free-floating sections were first blocked at room temperature for 1 h (PBS 1X, 0.1% of Triton X-100 and 3% of goat serum) and then were incubated with the primary antibodies (α-TH, 1:200, Millipore, Merck Life Science, Madrid, Spain, AB152; α-TH, 1:300, Sigma-Aldrich, Merck Life Science, Madrid, Spain, T2928; α-GFAP, 1:250, Dako, Agilent Technologies, Madrid, Spain, Z0334; α-GFAP, 1:250, Sigma, G3893; α-COX2, 1:200, Cayman Chemical, Ann Arbor, MI, USA, 160106, 160,106; α-TNFα, 1:200, Abcam plc, Cambridge, UK, ab1793) diluted in blocking solution at 4 °C for 24 h. After this, they were incubated for 1 h with the corresponding secondary antibody and 4′, 6-diamidino-2- phenylindole (DAPI; Calbiochem, Merck Life Science, Madrid, Spain 268298) for nuclear staining and diluted in blocking solution at room temperature. Some sections were stained with the fluorescent marker Tomato Lectin (1:150, Vector Labs, Newark, CA, USA, TL1176). Finally, sections were mounted with wet mounting medium (Vectashield; Vector Labs) and observed under an epifluorescence microscope (Nikon 90i).

### 2.14. Fluoro-Jade B Staining

This type of histochemical staining reveals degenerating neurons [24]. Brain sections, previously mounted on gelatinized slides, were immersed in absolute ethanol, followed by immersion in 70% ethanol and distilled water. Then, slides were incubated in 0.06% potassium permanganate for 15 min at room temperature. After this, sections were incubated in the staining solution (0.001% Fluoro-Jade B (Chemicon, Madrid, Spain, AG310) in acetic acid) for 30 min at room temperature and finally rinsed in distilled water, dried at room temperature, and mounted with DePeX (Serva). Images were acquired by confocal microscopy. To compare fluorescence signals from different preparations, confocal microscope settings were fixed for all samples within the same analysis group and adjusted to produce the optimum signal-to-noise ratio.

### 2.15. TUNEL (Free DNA End Marking by Biotinylated dUTPs)

DNA fragmentation in apoptotic cells was detected by marking the free DNA end with biotinylated dUTPs (TUNEL) using the in situ Cell Death Detection kit (Ref.11684817910; Sigma-Aldrich) and following the manufacturer’s instructions. Briefly, coronal brain sections containing SNpc were fixed with 4% paraformaldehyde for 20 min, and 3% H_2_O_2_ was used for endogenous peroxidase inactivation. Tissue was later permeabilized with 0.1% triton and 0.1% sodium citrate for 2 min at 4 °C, and free DNA ends were marked with biotinylated dUTPs in TdT buffer for 60 min. After that, sections were incubated with horseradish peroxidase (HRP)-conjugated streptavidin for 30 min at 37 °C. DNA breaks were observed using a 3-3′-diaminobendicina (DAB) chromogen at a final concentration of 50 μg/mL and H_2_O_2_. Brain sections treated with 2U of DNAse (Promega, Promega Biotech Ibérica, Alcobendas, Madrid, Spain, Ref.M610A) were used as positive controls for DNA fragmentation. Pictures were taken with a Nikon Eclipse 80i microscope coupled with a Nikon DS-Fi camera.

### 2.16. Cell Count Analysis

The numbers of dopaminergic neurons (TH-immunoreactive cells), astroglia (GFAP-positive cells), microglial cells (tomato-lectin-stained cells), Fluoro-Jade B and active caspase 3 marked cells as well as COX-2 and TNFα expressing cells in the SNpc were estimated. To that end, a modified stereological approach was used, as previously described [25]. Confocal images of serial coronal sections (30 μm) containing the entire SNpc (rostrocaudal extent) were acquired under an objective (×63) to avoid oversampling errors. The number of positive cells was counted in a 1:5 series of sections. The boundaries of the SNpc were determined with reference to internal anatomic landmarks [26]. Images were analyzed using computer-assisted image analysis software (Soft Imaging System Corporation, Lakewood, CO, USA). Four animals were used per group. The total number of positive cells for a particular marker in the SNpc was determined by multiplying the average number of labeled cells/section by the total number of 30-μm-thick sections containing the SNpc. Data are expressed as percentages to allow better understanding.

### 2.17. Statistical Analysis and Figures

Data were statistically analyzed using SPSS statistical software (IBM Corp., Armonk, NY, USA, Published in 2016. IBM SPSS Statistics for Windows, version 24.0. Armonk, NY, USA) through one-way analysis of variance (ANOVA), followed by a multiple comparison test with Bonferroni (if the data showed normality and homoscedasticity) or Games–Howell correction (if the data showed normality but not homoscedasticity) or the Kruskal–Wallis test, followed by Dunn’s multiple comparisons test (if data showed neither normality nor homoscedasticity). *p*-value < 0.05 was considered statistically significant. Figures were made with the Adobe Illustrator software (CS6, Adobe System Incorporated, San Jose, CA, USA).

## 3. Results

### 3.1. In Vivo Effect of LA

**Figure 1:**
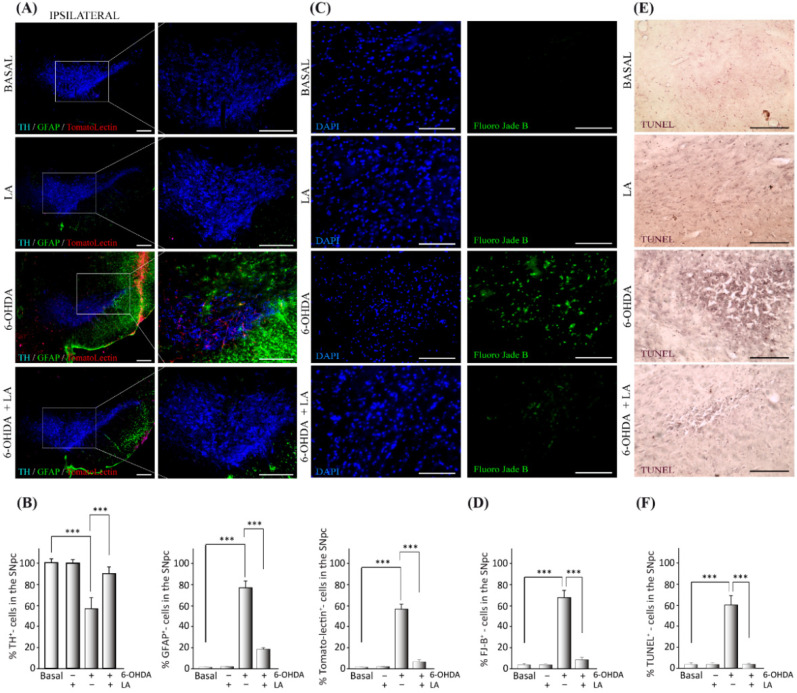
Neuroprotective effect of LA on a hemi-Parkinsonian animal model. Animals were injected unilaterally with 6-OHDA in the SNpc in combination or not with LA. The epifluorescence microscope images are ipsilateral coronal sections containing SNpc samples from the different experimental groups. (**A**) Representative images of the SNpc showing the presence of dopaminergic neurons labeled with tyrosine hydroxylase (TH, blue); astroglial cells stained with glial fibrillary acidic protein (GFAP, green) and Tomato lectin (red) as a marker for activated microglial cells. Scale bar, 200 μm. The right columns show greater magnification of the area delimited in the images in the left column (100 μm). (**B**) Quantification of the number of dopaminergic, astrocyte, and microglial cells shown in (A). (**C**) Analysis of the presence of degenerating neurons (green) in the SNpc measured using Fluoro-Jade B staining (DAPI was used as a nuclear stain). Scale bar, 100 μm. (**D**) Quantification of the number of Fluoro-Jade B (FJ-B) cells shown in (**C**). (**E**) Apoptotic neurons in the SNpc measured by the TUNEL assay. Scale bar, 100 μm. (**F**) Quantification of the number of TUNEL-positive cells in (**E**). The values in (**B**,**D**,**F**) represent the mean ± SD, expressed as the percentage of positive cells in the SNpc, given a particular marker, from three different experiments. There were four animals/experiment/experimental group and five independent sections per animal. *** *p* < 0.001.

**Figure A1:**
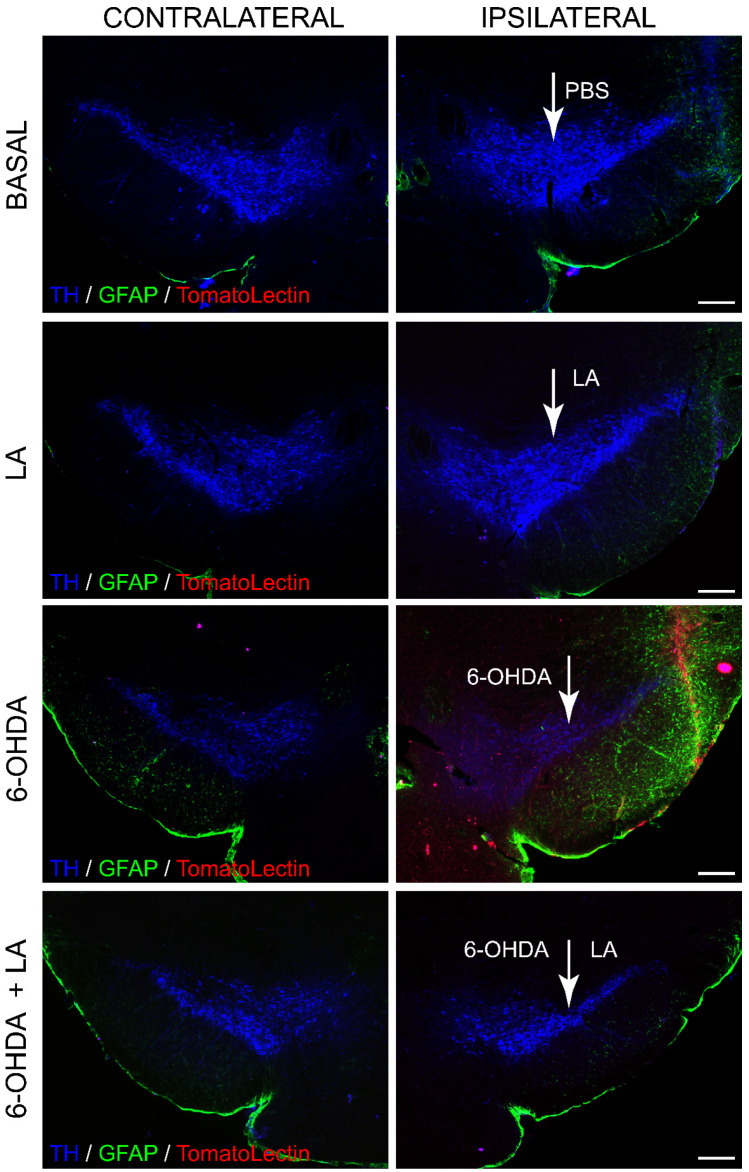
Neuroprotective and anti-inflammatory effect of LA on a hemiparkinsonian animal model. Representative images of coronal sections of the mouse SNpc showing the presence of dopaminergic neurons (TH, blue); astroglial cells labeled with glial fibrillary acidic protein (GFAP, green) and Tomato lectin (red) as a marker of activated microglial cells, both in the contralateral (not injected) and ipsilateral (injected) hemisphere. Scale bar, 200 μm.

Previous studies linked LA-stimulating activity with LD biogenesis and autophagic flux [18,19], suggesting a neuroprotective and anti-inflammatory effect of LA against neurodegenerative diseases. Hence, we first assessed whether LA does indeed have neuroprotective and anti-inflammatory effects in vivo. To this end, adult mice were lesioned with 6-OHDA intracerebrally and unilaterally in the SNpc in the presence or absence of LA. 6-OHDA injection into the SNpc of rodents induces dopaminergic cell loss and glial activation [23]. We evaluated the magnitude of dopaminergic cell loss and glial activation in the SNpc by immunohistochemistry analysis. Figure 1A,B and Figure A1 show that the hemisphere treated with 6-OHDA presented high levels of dopaminergic neuron degeneration together with the activation of astrocytes and microglial cells in contrast to the nontreated hemisphere. Interestingly, LA co-administration was able to prevent the neurodegeneration and neuroinflammation induced by 6-OHDA (Figure 1A,B and Figure A1). LA injection by itself did not result in cytotoxic or proinflammatory effects, because the signals of dopaminergic neurons and activated glial cells were similar in both hemispheres (Figure 1A,B and Figure A1). Next, we studied the neuroprotective effect of LA in vivo through TUNEL and Fluoro-Jade B staining. These markers identify neuron apoptosis and neurodegeneration, respectively. Fluoro-Jade B staining and quantification showed that LA significantly decreased the neurodegeneration present in the in vivo PD model (Figure 1C,D). TUNEL staining also demonstrated that LA strongly reduced apoptotic neuron signals in the in vivo PD model (Figure 1E,F). These studies indicate a near to complete neuroprotective effect of LA in an in vivo PD model.

**Figure 2:**
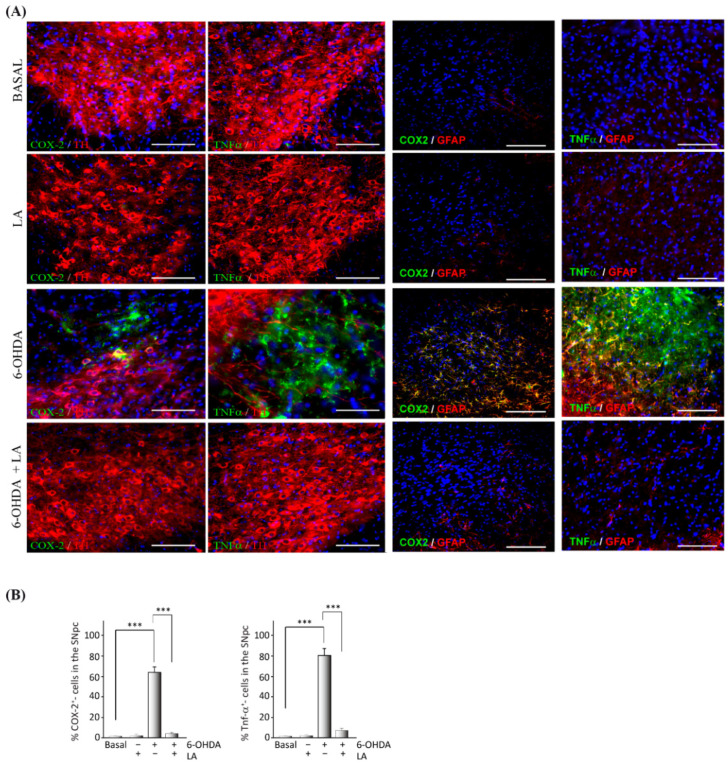
Anti-inflammatory effect of LA on a hemi-Parkinsonian animal model. Animals were injected unilaterally with 6-OHDA in the SNpc in combination or not with LA. The epifluorescence microscope images are ipsilateral coronal sections containing the SNpc from the different experimental groups. (**A**) Immunohistochemical identification of proinflammatory molecules (green) in the SNpc: cyclooxygenase 2 (COX-2) and tumor necrosis factor-alpha (TNFα). Dopaminergic neurons are labeled with a tyrosine hydroxylase antibody (TH, red), astroglial cells are marked with glial fibrillary acidic protein (GFAP, red), and nuclei are marked with DAPI. Scale bar, 100 μm. (**B**) The quantification of COX-2 and TNFα positive cells through the SNpc is shown. The values represent the mean ± SD, expressed as percentages, from three different experiments. There were four animals/experiment/experimental group and five independent sections per animal. *** *p* < 0.001.

Next, we studied the anti-inflammatory effect of LA in the in vivo PD model through immunohistochemical assays against the pro-inflammatory markers cyclooxygenase-2 (COX-2) and tumor necrosis factor α (TNF-α). The presence of COX-2 and TNF-α in the SNpc (localized with the TH marker) significantly decreased in the in vivo PD model treated with LA compared with nontreated animals (Figure 2). This decrease in proinflammatory markers was accompanied by reduced signals from reactive astrocytes.

### 3.2. In Vitro Effect of LA

**Figure A2:**
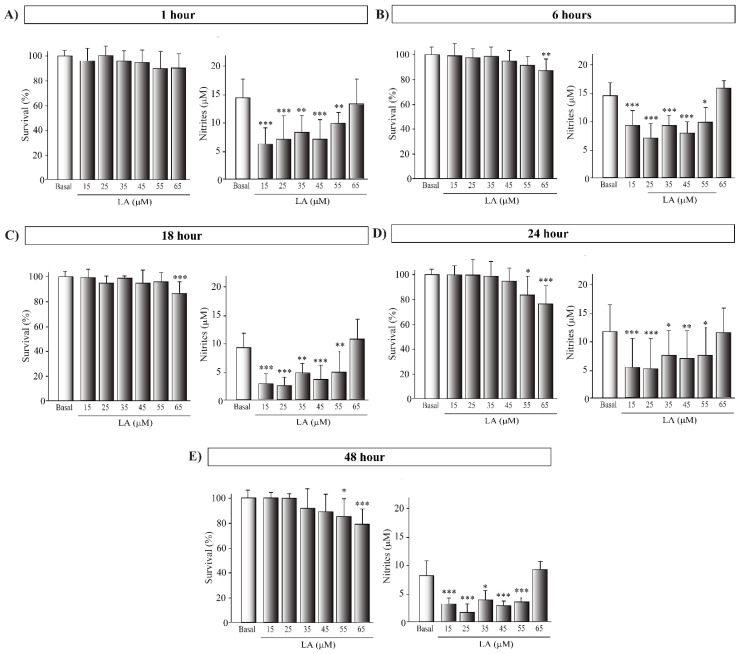
Cytotoxic effect of LA on SH-SY5Y cell line. Determination of cell viability by MTT assay and nitrites concentration by Griess assay of the human cell line SH-SY5Y treated or not with LA. Each graph corresponds to different LA concentrations and preincubation times: 1 (**A**), 6 (**B**), 18 (**C**), 24 (**D**) and 48 (**E**) hours. The mean of the normalized cell viability percentage of the different treatment groups to the control group is shown. The mean of nitrites concentration of the different treatment groups is shown. Results were obtained from three independent experiments with six repetitions each per experimental group, and the error bars correspond to ± SD. *** *p* < 0.001; ** *p* < 0.01; * *p* < 0.05 versus control group.

We next used an in vitro approach to decipher the molecular mechanisms underlying the neuroprotective and anti-inflammatory effects observed in vivo with LA treatment on a hemi-Parkinsonian animal model (Figure 1 and Figure 2). We first studied the possible cytotoxic and pro-inflammatory effects of LA at different concentrations (15, 25, 35, 45, 55, and 65 μM) and preincubation times (1, 6, 18, 24, and 48 h) in the SH-SY5Y cell line. This cell line provides an unlimited supply of cells of human origin with similar biochemical characteristics to human dopaminergic neurons [27]. We found that treatment with LA did not significantly decrease the cell viability within the range of 15 to 45 μM at any of the preincubation times tested (Figure A2). Nevertheless, treatment with 55 μM of LA with pre-incubation for at least 24 h caused a significant decrease in the viability of the SH-SY5Y cells (Figure A2). We also studied the possible proinflammatory effect of LA by the Griess assay, which revealed that LA causes a significant reduction in nitrite production at most concentrations and preincubation times tested (Figure A2).

**Figure 3:**
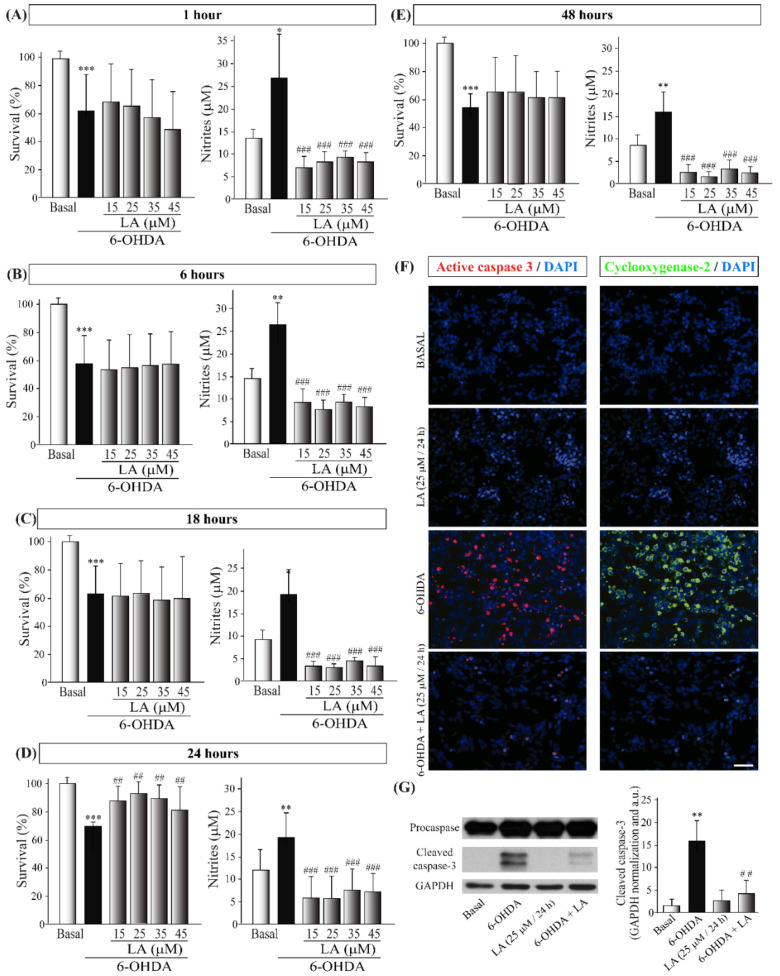
In vitro neuroprotective and anti-inflammatory effects of LA on the SH-SY5Y PD model. The human cell line SH-SY5Y was pre-exposed to different concentrations of LA (15, 25, 35, 45, 55, and 65 μM) for 1 (**A**), 6 (**B**), 18 (**C**), 24 (**D**), or 48 h (**E**) before incubation with 35 µM 6-OHDA for 18 h. The cell viability was measured by MTT assay, and determination of the nitrite concentration was evaluated by Griess reaction. Each bar represents the mean ± SD of three independent experiments with 6 replicates per experimental group. Data were analyzed using one-way analysis of variance (ANOVA) followed by the Games–Howell correction in some cases and the Kruskal–Wallis test followed by Dunn’s multiple comparisons test in other cases. *** *p* < 0.001, ** *p* < 0.01, * *p* < 0.05 versus control group; ##: *p* < 0.01 and ###: *p* < 0.001 versus 6-OHDA-treated cultures. (**F**) Immunofluorescence images (epifluorescence microscope) of the apoptotic protein active caspase-3 (red) and the proinflammatory factor cyclooxygenase-2 (COX-2, green). Dapi was used as a nuclear marker. Representative results of three independent experiments are shown. Calibration bar: 100 µm. (**G**) Representative Western blot and quantification showing the expression of cleaved caspase-3 normalized by GAPDH. Data are the results of three independent experiments, and the error bars correspond to ± SD. Data were analyzed using a one-way analysis of variance (ANOVA) followed by a Tukey correction. ** *p* <0.01 in comparison with the basal group and ## *p* < 0.01 in comparison with the 6-OHDA group.

Then, we studied the neuroprotective and anti-inflammatory effects of LA in the well-established in vitro model of PD, the SH-SY5Y cell line treated with 6-OHDA [23]. The concentrations of LA were 15, 25, 35, and 45 μM, and the preincubation times were 1, 6, 18, 24, and 48 h (Figure 3). The viability assays showed that LA significantly prevents the decrease in cell viability caused by 6-OHDA at concentrations of 15, 25, and 35 μM added 24 h before the 6-OHDA treatment (Figure 3D). On the other hand, the Griess assay showed that LA significantly prevents an increase in the nitrite concentration (caused by 6-OHDA) at all concentrations and preincubation times tested (Figure 3A–E). These results indicate that, besides its protective effect on cell viability, LA also exerts a potent anti-inflammatory effect. Given these data, we used an LA concentration of 25 μM and a preincubation time of 24 h for the following experiments.

To further confirm these effects, we performed immunocytochemical and Western blot analyses in the SH-SY5Y cells treated with 6-OHDA and/or LA using cleaved caspase-3 and COX-2 antibodies. As shown in Figure 3F,G, treatment with LA strongly prevented the increases in apoptotic cell death and inflammation present in the SH-SY5Y cell line treated with 6-OHDA, confirming the above-presented data.

### 3.3. Mechanism of Action of LA Regulation of Autophagic Flux and Lipid Droplet Levels

**Figure 4:**
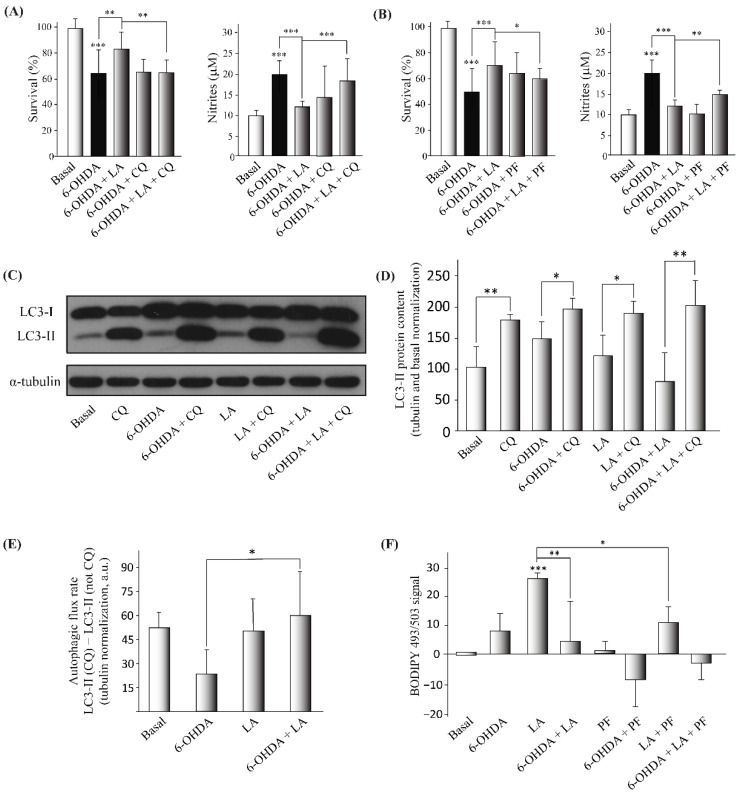
Regulation of the autophagic flux and lipid droplet levels by LA in the SH-SY5Y PD model. The human cell line SH-SY5Y was exposed to 35 µM 6-OHDA in the presence or not of LA, PF, and CQ. (**A**,**B**) Cell viability was measured by MTT assay, with LA, 6-OHDA, CQ, or PF added or not. The nitrite concentration was evaluated by Griess reaction under same conditions. Each bar represents the mean ± SD of three independent experiments with 6 replicates per experimental group. Data were analyzed using one-way analysis of variance (ANOVA) followed by the Games–Howell correction in some cases and the Kruskal–Wallis test followed by Dunn’s multiple comparisons test in other cases. *** *p* < 0.001, ** *p* < 0.01, * *p* < 0.05. (**C**,**D**) Representative Western blot and quantification showing the expression of LC3-II normalized by tubulin and by the control levels (%). Data represent the results of three independent experiments, and the error bars correspond to ± SD. Data were analyzed using one-way analysis of variance (ANOVA) followed by the Games–Howell correction. * *p* < 0.05 and ** *p* < 0.01 (if not indicated, it is with respect to the control). (**E**) The graph shows the autophagic flux rate (arbitrary units) of the different experimental groups. These measurements represent the subtraction of the LC3-II levels (arbitrary units normalized by tubulin) in the non-CQ group from those in the CQ group. The mean ± SD correspond to four independent experiments. Data were analyzed using the ANOVA test followed by Tukey’s multiple comparisons test. * *p* < 0.05 in comparison with 6-OHDA. (**F**) Determination of the BODIPY 493/503 signal was done by flow cytometry in the SH-SY5Y cell line treated or not with LA, 6-OHDA, and/or PF. The graph shows the mean value of the BODIPY 493/503 signal for the different treatment groups normalized by the control group. The results correspond to 6 independent experiments per group, and the error bars show ±SD. Data were analyzed using the Kruskal–Wallis test followed by Dunn’s multiple comparisons test. *** *p* < 0.001 (if not indicated, it is with respect to the control), ** *p* < 0.01; * *p* < 0.05.

**Figure A3:**
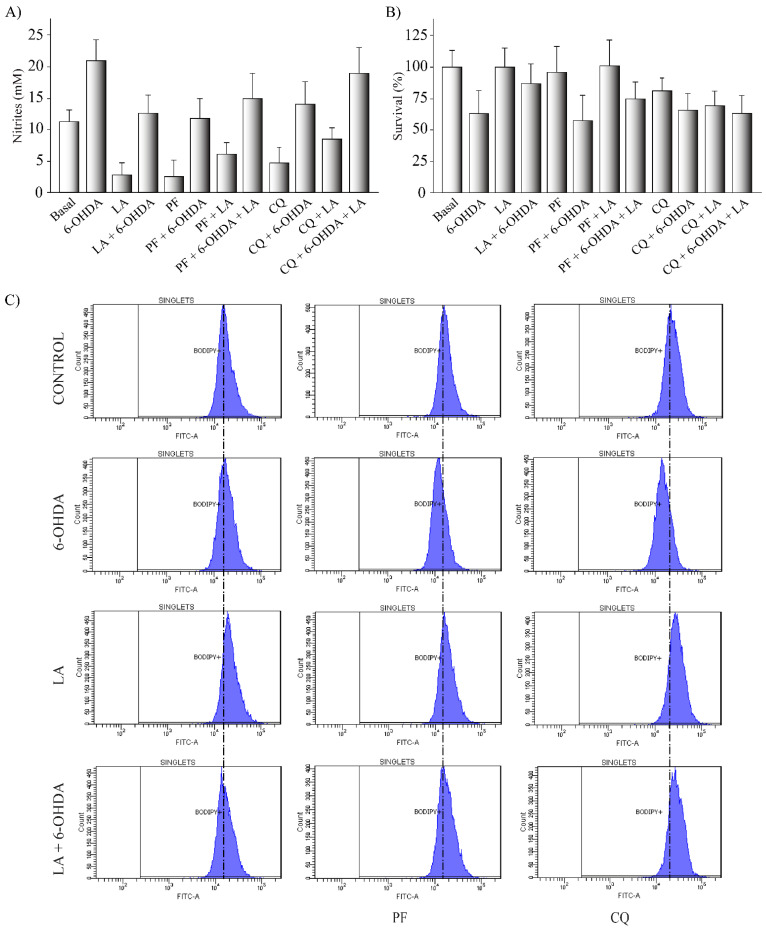
(**A**,**B**) The human cell line SH-SY5Y was exposed to 35 µM 6-OHDA in the presence or not of LA, PF, and CQ. The cell viability was measured by MTT assay and determination of nitrites concentration was evaluated by Griess reaction. Each bar represents the mean ± SD of three independent experiments with 6 replications each per experimental group. (**C**) BODIPY 493/503 flow cytometry experiments on SH-SY5Y PD model. Representative histograms of flow cytometry experiments with the SH-SY5Y cells marked with BODIPY 493/503 (FITC-A signal) and treated or not with LA, 6-OHDA, PF, and/or CQ. The results correspond to 6 independent experiments per group.

After confirming the neuroprotective and anti-inflammatory effects of LA in the in vitro model of PD, we aimed to decipher its mechanism of action. Due to previous evidence of the effect of some fatty acids on LD dynamics and autophagy [18,19], we first checked whether LA treatment had any effect on the autophagic flux and LD dynamics. As before, we performed cell viability and Griess assays, but we added the autophagy inhibitor chloroquine (CQ) to test the involvement of autophagy in the effect of LA. The results obtained suggest that the blocking of the autophagic flux significantly reverses the protective effect of LA (Figure 4A and Figure A3). Then, we performed an LC3 turnover assay by Western blot using the autophagy inhibitor CQ (the difference in the level of LC3-II in the same group when autophagic flux is inhibited versus when it is not is considered the working autophagic flux). In this way, we measured the level of the protein LC3-II (a marker of autophagosomes) under different experimental conditions. As shown in Figure 4C–E, 6-OHDA treated cells presented a tendency to impair the autophagic flux, as previously described [28]. Indeed, these 6-OHDA treated cells tended to accumulate more LC3-II than the control both when the autophagic flux was inhibited and when it was not. These results suggest that 6-OHDA may stimulate autophagic flux in the SH-SY5Y cells, but it may also lead to incorrect degradation of the autophagosomes, hence impairing the autophagic flux. Nonetheless, LA was associated with a significant reduction of LC3-II accumulation caused by 6-OHDA when autophagy flux was active (without CQ treatment). On the other hand, there were no differences between 6-OHDA and LA plus 6-OHDA treated cells when autophagic flux was inhibited (CQ treated groups). These results suggest that treatment with LA improves the autophagy flux, probably by ameliorating the autophagosome degradation blockade provoked by 6-OHDA.

Next, we studied the effect of LA treatment on LD dynamics to analyze whether LDs are involved in the mechanism of action of LA. For this purpose, we used PF-06424439 (PF), a specific inhibitor of diacylglycerol acyltransferase 2 (DGAT-2) and hence an inhibitor of LD biogenesis [9,19] and once again performed cell viability and Griess assays. Our results show that the inhibition of LD biogenesis partially reverted the neuroprotective and anti-inflammatory effects of LA in the PD model (Figure 4B and Figure A3). To further study the effect of LA treatment on LD dynamics, we also measured the levels of LDs by flow cytometry with the LD marker BODIPY 493/503. Figure 4F and Figure A3 show that LA caused a significant increase in the level of LDs in the SH-SY5Y cell line under basal conditions, and this increase was partially reduced when LA treatment was combined with PF. Moreover, the increase in the level of LDs by LA treatment was also significantly reduced when cells were also treated with 6-OHDA. These results indicate that LA causes an increase in LDs in a DGAT-2 partially dependent way in the SH-SY5Y cell line. However, the increase in the level of LDs with LA treatment was significantly reduced when the cells were also treated with 6-OHDA.

### 3.4. Lipid Droplet Recycling Mechanism

**Figure 5:**
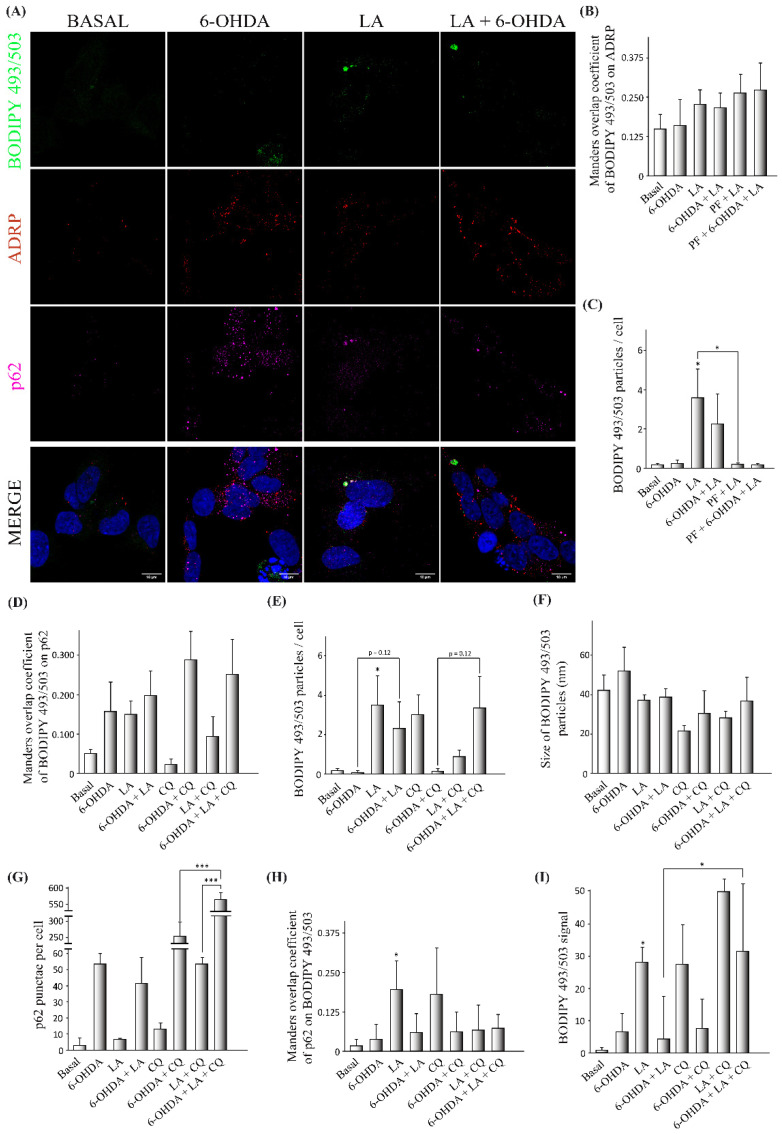
Effect of LA on lipophagic flux and LD dynamics in the SH-SY5Y PD model. The human cell line SH-SY5Y was exposed to 35 µM 6-OHDA in the presence or not of LA, PF, and CQ. (**A**) Representative maximum intensity projection of confocal images showing the immunocytochemical analysis with the BODIPY 493/503 marker (green), ADRP (red), and p62 (magenta) on SH-SY5Y cells. (**B**) Colocalization analysis of BODIPY 493/503 on ADRP with the Manders overlap coefficient. (**C**) Analysis of BODIPY 492/503 particles per cell. (**D**) Colocalization analysis of BODIPY 493/503 on p62 with the Manders overlap coefficient. (**E**) Analysis of BODIPY 492/503 particles per cell. (**F**) Analysis of the size of BODIPY 492/503 particles per cell. (**G**) Analysis of p62 punctae per cell. (**H**) Colocalization analysis of p62 on BODIPY 493/503 with the Manders overlap coefficient. Graphs show the mean ± SD of 6 independent experiments per group. Data were analyzed by one-way analysis of variance (ANOVA), followed by a multiple comparison test with Bonferroni in some cases and one-way analysis of variance (ANOVA) followed by the Games–Howell correction in other cases. *** *p* < 0.001; * *p* < 0.05 vs. control or between two specific groups when indicated). (**I**) Determination of the BODIPY 493/503 signal by flow cytometry. The mean BODIPY 493/503 signal normalized by the control for the different treatment groups is shown. The results correspond to 6 independent experiments per group. Data were analyzed using the Kruskal–Wallis test followed by Dunn’s multiple comparisons test. * *p* < 0.05 vs. control or between two specific groups when indicated.

**Figure A4:**
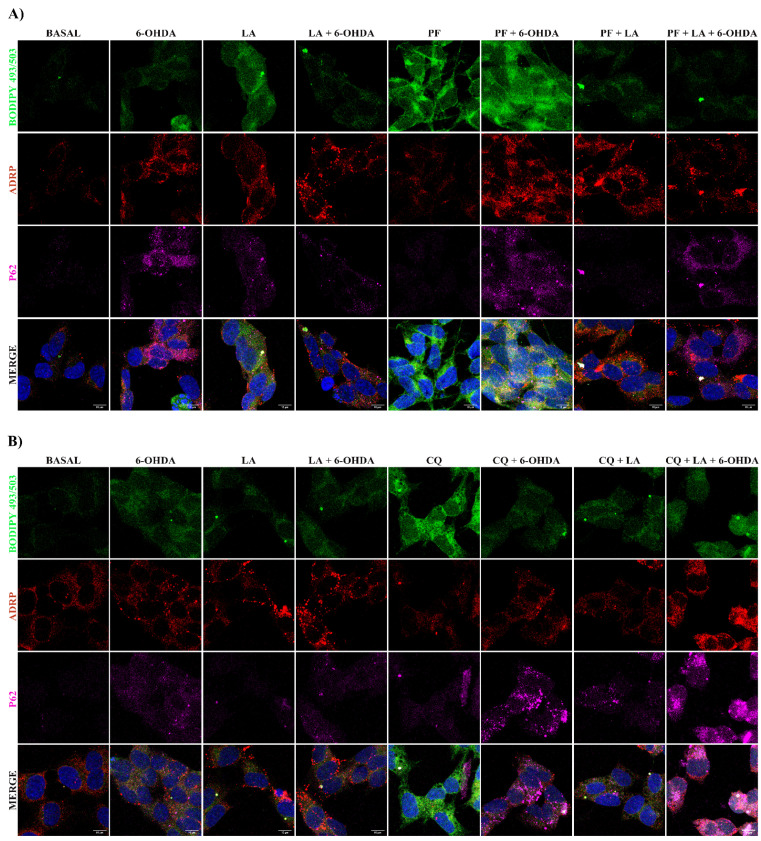
Study of the LA effect on lipophagic flux and LDs dynamics on SH-SY5Y PD model. Representative maximum intensity projection of confocal images with the BODIPY 493/503 marker (green), ADRP (red), and p62 (magenta) from the SH-SY5Y cell line treated or not with LA, 6-OHDA, PF (**A**), and/or CQ (**B**) are shown.

Given the previous results, we further explored the possible involvement of autophagic flux and LDs in the effect of LA through the proposed LD recycling mechanism. For this purpose, we performed immunocytochemical analyses on the SH-SY5Y PD model with BODIPY 493/503, adipose differentiation-related protein (ADRP, a surface protein of LDs), and sequestosome-1 (SQSTM1/p62, an autophagy receptor with important functions in lipophagy [29,30]) with the final goal of understanding the effect of LA on the level and size of LDs, the association of ADRP with LDs, the lipophagic levels, and the autophagic flux (Figure 5 and Figure A4).

For this purpose, we used Manders Overlap Coefficients (MOC, M1 and M2) to evaluate the colocalization of different markers, with M1 determining the portion of the signal from the first fluorophore that colocalized with the second fluorophore and M2 exchanging the first and second fluorophores. First, we observed that the LDs detected by BODIPY 493/503 in the SH-SY5Y cell line partially colocalized with the ADRP signal, validating the BODIPY 493/503 signal as a marker of LDs (Figure 5A,B). We also observed that LA causes a significant increase in the LD level in the SH-SY5Y cell line under basal conditions, and a trend for this effect was shown when these cells were treated with 6-OHDA (Figure 5C), although LA treatment did not change the mean size of the LD particles (Figure 5F). Interestingly, this increase in the number of LDs caused by LA was significantly abolished when DGAT2 was inhibited by PF treatment in the SH-SY5Y cell line (Figure 5C), suggesting a reduction in the biogenesis of LDs. Moreover, the inhibition of DGAT2 was also associated with a trend toward a reduction in the level of LDs in the SH-SY5Y cells treated with 6-OHDA + LA (Figure 5C). We also analyzed the MOC values of BODIPY 493/503 on p62, a proxy for LDs degraded by lipophagy. Although the results did not reach significance, the SH-SY5Y cells treated with 6-OHDA or LA showed a trend towards having more LDs colocalized with p62 (Figure 5D).

The next step was to analyze whether the inhibition of autophagy flux by CQ could have an impact on the LD level under the different experimental conditions. CQ treatment caused an expected accumulation of LDs in the SH-SY5Y cells (Figure 5E). Interestingly, 6-OHDA + CQ treatment was not associated with an apparent accumulation of LDs, but when LA was added, this fatty acid seemed to promote the accumulation of LDs previously observed to be altered by 6-OHDA (Figure 5E). LA + CQ treatment did not increase the level of LDs compared with LA treatment alone (Figure 5E). These results suggest that LA increases the degradation of LDs by lipophagy in SH-SY5Y cells treated with 6-OHDA.

Next, in order to assess the effect of LA on the total autophagic flux, we measured the p62 punctae present in groups treated or not with CQ (Figure 5G). We found a significant increase in p62 punctae in cells treated with 6-OHDA + LA + CQ in comparison with cells treated only with 6-OHDA + CQ. In the same way, we observed an increased number of p62 punctae in cultures treated with LA + CQ compared with those treated only with CQ. These results indicate that LA increases the autophagic flux in the SH-SY5Y cells treated with 6-OHDA.

Finally, we used the MOC value of p62 on BODIPY 493/503 as a measure of the p62 proportion involved in processes related to LD dynamics (such as degradation by lipophagy) for every experimental condition (Figure 5H). LA significantly increased the portion of p62 colocalized with LDs under control conditions. The redistribution of p62 in the PD model was not as strong as under control conditions, which may have been due to the increased p62 autophagic flux present in these 6-OHDA treated cells (Figure 5G). Our results suggest that LA redistributes p62 (and probably the autophagic flux or lipolysis) to LDs in control SH-SY5Y cells.

To further clarify the effect of LA on the degradation of LDs, we measured the level and intensity of LDs by flow cytometry using the marker BODIPY 493/503 under different experimental conditions in the SH-SY5Y cells (Figure 5I and Figure A3). This study shows that treatment with LA and CQ, under both control conditions and in 6-OHDA-treated cells, was associated with a clear tendency toward increased levels of LDs in comparison with cells treated only with LA. In summary, our results suggest that LA increases lipophagy under basal conditions, predominantly in 6-OHDA-treated SH-SY5Y cells.

### 3.5. Regulation of Lipid Peroxidation by LA

**Figure 6:**
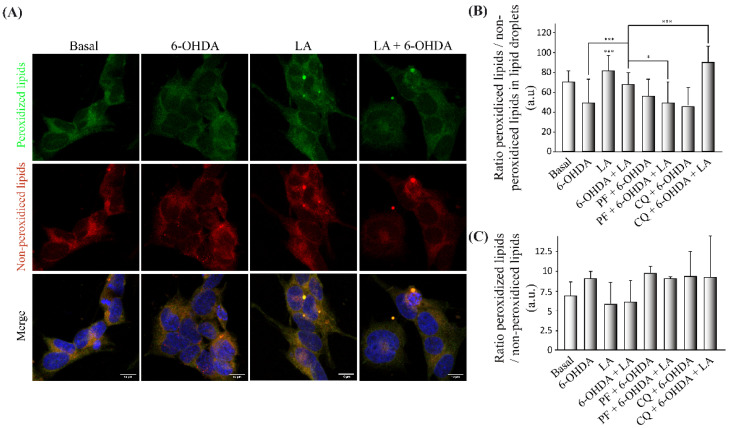
Regulation of lipid peroxidation by LA in the SH-SY5Y PD model. (**A**) Representative maximum intensity projection of confocal images showing the immunocytochemical assay with the BODIPY 581/591 C11 marker. Peroxidized lipids are shown in green, and nonperoxidized lipids are shown in red on cells treated or not with LA and 6-OHDA. (**B**) Quantification of the peroxidized lipids/nonperoxidized ratio in the LDs of the different experimental groups. The mean ± SD of 6 experiments per group is shown. Data were analyzed using one-way analysis of variance (ANOVA) followed by a multiple comparison test with Bonferroni. *** *p* < 0.001; * *p* < 0.05 (if not indicated, it is for the control). (**C**) Determination of the BODIPY 581/591 C11 signal by flow cytometry on the SH-SY5Y cell line treated or not with LA, PF, CQ, and/or 6-OHDA. The graph shows the mean of the peroxidized/nonperoxidized lipids ratio obtained from the BODIPY 581/591 c11 signals of the different treatment groups. The results correspond to the mean ± SD of 4 independent experiments per group. Data were analyzed by one-way analysis of variance (ANOVA), followed by the Games–Howell correction.

**Figure A5:**
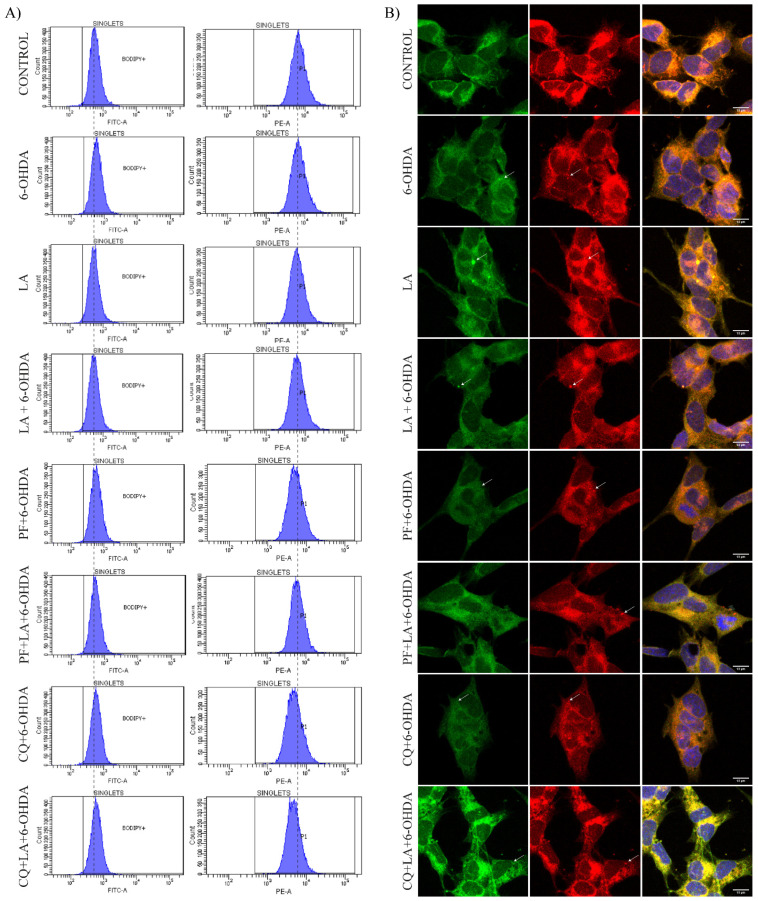
(**A**) BODIPY 581/591 C11 flow cytometry experiments on SH-SY5Y PD model. Representative histograms of flow cytometry experiments with the SH-SY5Y cells marked with BODIPY 581/591 C11 and treated or not with LA, 6-OHDA, PF, and/or CQ. The left column corresponds to the FITC-A signal (peroxidized lipids), and the right column corresponds to the PE-A signal (non-peroxidized lipids). The results correspond to 6 independent experiments per group. (**B**) Representative maximum intensity projection of confocal images showing the immunocytochemical assay with BODIPY 581/591 C11 marker. It shows peroxidized lipids in green and non-peroxidized lipids in red on cells treated or not with LA, PF, CQ, and/or 6-OHDA.

After obtaining some hints regarding the LD recycling mechanism by LA, we further studied its effect on the lipid peroxidation LD state [13,15,16]. For this purpose, we performed an immunocytochemical analysis with the marker BODIPY 581/591 C11 (a marker of peroxidized and nonperoxidized lipids at distinct wavelengths) in the in vitro model of PD with inhibitors. Specifically, we measured the ratio of peroxidized (green)/nonperoxidized lipids (red) in the particles (LDs) observed in the images of the cells. Figure 6A,B and Figure A5B show a tendency for the ratio of peroxidized lipids to decrease in the LDs of cells treated with 6-OHDA compared with basal cells. Regarding the effect of LA, this fatty acid caused a significant increase in the lipid peroxidation ratio of LDs in both control conditions and in the PD model. Furthermore, the addition of the inhibitors (PF and CQ) to the PD model treated with LA caused significant changes in the lipid peroxidation ratio. PF prevented the increase in the lipid peroxidation ratio caused by LA. On the contrary, CQ significantly increased this lipid peroxidation ratio in the PD model treated with LA.

Additionally, we measured the total lipid peroxidation ratio, not only the LD particles. For this purpose, we performed the FACS analysis to quantify the total lipid peroxidation ratio using BODIPY 581/591 C11. The results obtained (Figure 6C and Figure A5A) show that, as expected, the lipid peroxidation ratio was higher in SH-SY5Y cells treated with 6-OHDA in comparison with those exposed to control conditions. Moreover, LA treatment caused a decrease in the total peroxidation ratio under control conditions and 6-OHDA treatment conditions. This decrease in the peroxidation ratio caused by LA was reversed by both inhibitors (PF and CQ).

## 4. Discussion

Our study demonstrates LA’s neuroprotective and anti-inflammatory effects using in vitro and in vivo models of PD. LA prevented the neurodegeneration of dopaminergic neurons in the SNpc of 6-OHDA damaged mice (Figure 1 and Figure 2) and the death of the SH-SY5Y cell line after damage with 6-OHDA (Figure 3). Additionally, LA prevented the neuroinflammation presented in both in vitro and in vivo models (Figure 1, Figure 2 and Figure 3). Furthermore, our study shows, for the first time, that the possible mechanism of action for LA in an in vitro PD model is through increasing the genesis of LDs and lipophagic flux to achieve an antioxidant effect. Further experiments are warranted to demonstrate the participation of lipids, lipid droplets, and autophagy/lipophagy in the pathogenesis of neurodegenerative diseases as well as the possible use of their regulation to treat these pathologies [7,31,32].

Regarding the possible mechanism of action of LA, we demonstrated that this fatty acid can induce the genesis of LDs under basal conditions (Figure 4 and Figure 5). This stimulation of the genesis of LDs by LA is dependent (at least in part) on the DGAT-2 enzyme (Figure 4 and Figure 5). However, the specific involvement of DGAT-2, as well as that of the DGAT-1 enzyme, needs to be verified in future studies. In this regard, the possible action mechanism of other fatty acids through lipid droplets biogenesis has been studied. Nakajima et al. [19] showed that oleic acid induces the genesis of lipid droplets in astrocytes, achieving significant stimulation of their biogenesis after 24 h of treatment. This is the same amount of time that it takes for LA to achieve its best neuroprotective effect. Additionally, they verified that this lipid droplet genesis is dependent on DGAT-2 (although it is more dependent on DGAT-1). Thus, they found that oleic acid esterifies in triacylglycerols, so it is not unreasonable to think that the same could happen with LA in the SH-SY5Y cell line.

However, the PD model treated with LA did not show an increase in the level of LDs. This difference was probably due to the degradation of LDs generated by LA. Indeed, we observed a possible induction of lipophagy (triggered by LA), which could underlie the decrease in the LD levels in SH-SY5Y cells treated with LA and 6-OHDA (Figure 4 and Figure 5). Although we also observed a tendency of LA to induce lipophagy in a basal situation (Figure 4 and Figure 5), its induction by LA mainly occurred in the PD model (cellular stress). However, more studies are needed to analyze other possible mechanisms for LD degradation (such as lipolysis) and to prevent possible side effects of the treatment. Yang et al. [33] proved that LA stimulates autophagic flux and an antioxidant response in hepatocytes and demonstrated the presence of a positive feedback loop between autophagy induction and antioxidant response by LA, which may coincide with our proposed “LD’ recycling mechanism”.

Regarding this antioxidant response of LA, we observed that LA caused an increase in the oxidative state of LDs and a possible decrease in the lipid peroxidation state of the cells (Figure 6) under SH-SY5Y basal conditions and in the PD model. Thus, LDs may sequester the oxidative stress present in the PD model. In this way, the inhibition of DGAT-2 with PF prevented the peroxidation of LD and produced a lipid peroxidation state improvement for LA in the SH-SY5Y PD model. Moreover, inhibition of the autophagic flux with CQ increased the peroxidation of LD and improved the lipid peroxidation state in the SH-SY5Y PD model treated with LA. These results show the importance of the stimulation of LD biogenesis by LA for its oxidant scavenger effect and to stimulate autophagic/lipophagic flux by LA to remove the accumulation of peroxidized LD. Given these data, it is necessary to verify the exact mechanism by which LDs sequester free radicals, given that we already have previous indications of the possible existence of this mechanism [34,35]. Additionally, other studies have observed the ability of LDs to protect different nerve cell types from oxidative stress [13,15,16].

Thus, our proposed mechanism of action, “LD recycling” (increasing genesis as well as degradation), may be a protective mechanism against lipocytotoxity (which causes a high level of oxidative stress) that gets rid of excess lipids. The accumulation of altered LDs in neural cell types can lead to pathological situations [12,31,36,37]. However, this mechanism could reduce the oxidative stress present in neurodegenerative diseases with specific concentrations of fatty acids (as has been shown in this study). This therapeutic strategy may have positive effects on the cellular oxidative state [13,15,16], proteostasis [6,32], the stability of the membranes [34], etc. Thus, our study increases the body of knowledge regarding the genesis of LDs, their antioxidant capacity, and the participation of lipophagy in a neuroprotective physiological process using in vitro and in vivo PD models. On the other hand, Cui et al. [38] recently demonstrated an extracellular flux of fatty acids due to lipophagy through exocytosis that could explain the future of LDs degraded by lipophagy found in this study. Furthermore, this evidence may relate to recent research showing a lipid metabolic coupling between neurons and astrocytes [13,14], whose failure is related to the presence of Apolipoprotein E4 and its alterations (the principal risk factor for Alzheimer’s disease) [14,31]. Consequently, these cellular effects of LA may be part of a broader physiological mechanism of action against lipocytoxicity. This mechanism could participate in the pathogenesis of neurodegenerative diseases and could be used as a novel treatment for these disorders. A limitation of the in vitro model used in this study is the partially oncogenic phenotype of the SH-SY5Y cells. Hence, carrying out future experiments in cultures that include all neural cell lines, such as mesencephalic organoids [39], is needed to allow the analysis of the relationship between neurons and glial cells on primary cultures and to verify our results.

In summary, the results presented here corroborate the neuroprotective and anti-inflammatory effects of LA against PD and suggest, for the first time, a new mechanism of action of LA for the treatment of neurodegenerative disorders, which underlies LDs, lipophagy, and oxidative stress processes. Thus, our study opens the door for further research on the use of coordinated regulation of the genesis of lipid droplets and lipophagy to regulate oxidative stress as a new therapeutic strategy against these diseases.

## Data Availability

The data that support the findings of this study are available from the corresponding author upon reasonable request.
